# Development and validation of a measure of health literacy in the UK: the newest vital sign

**DOI:** 10.1186/1471-2458-13-116

**Published:** 2013-02-07

**Authors:** Gill Rowlands, Nina Khazaezadeh, Eugene Oteng-Ntim, Paul Seed, Suzanne Barr, Barry D Weiss

**Affiliations:** 1 London South Bank University, London, UK; 2 Guy’s and St Thomas’ NHS Foundation Trust, London, UK; 3 Kings College London, London, UK; 4 University of Arizona, Tucson, AZ, USA

**Keywords:** Literacy, Health literacy, Nutrition label, Measure

## Abstract

**Background:**

Health literacy (HL) is an important public health issue. Current measures have drawbacks in length and/or acceptability. The US-developed Newest Vital Sign (NVS) health literacy instrument measures both reading comprehension and numeracy skills using a nutrition label, takes 3 minutes to administer, and has proven to be acceptable to research subjects. This study aimed to amend and validate it for the UK population.

**Methods:**

We used a three-stage process; (1) a Delphi study with academic and clinical experts to amend the NVS label to reflect UK nutrition labeling (2) community-based cognitive testing to assess and improve ease of understanding and acceptability of the test (3) validation of the NVS-UK against an accepted standard test of health literacy, the Test of Functional Health Literacy in Adults (TOFHLA) (Pearson’s r and the area under the Receiver Operating Characteristic (ROC) curve) and participant educational level. A sample size calculation indicated that 250 participants would be required. Inclusion criteria were age 18–75 years and ability to converse in English. We excluded people working in the health field and those with impaired vision or inability to undertake the interview due to cognitive impairment or inability to converse in English.

**Results:**

In the Delphi study, 28 experts reached consensus (3 cycles). Cognitive testing (80 participants) yielded an instrument that needed no further refinement. Validation testing (337 participants) showed high internal consistency (Cronbach’s Alpha = 0.74). Validation against the TOFHLA demonstrated a Pearson’s r of 0.49 and an area under the ROC curve of 0.81.

**Conclusions:**

The NVS-UK is a valid measure of HL. Its acceptability and ease of application makes it an ideal tool for use in the UK. It has potential uses in public health research including epidemiological surveys and randomized controlled trials, and in enabling practitioners to tailor care to patient need.

## Background

Health literacy (HL) is defined as ‘the cognitive and social skills that determine the motivation and ability of individuals to gain access to, understand and use information in ways that promote and maintain good health’ [1,2]. It is recognised as an important cause of health inequalities in industrialised nations such as the UK, US, Canada, and Australia [2-6]. HL is a complex concept, with multiple components [7]. The ability to understand both language and numbers in health contexts are core competencies. Health literacy is associated with educational status and other social determinants of health such as ethnicity and socio-economic status, but has an association with health and long-term conditions that persists even when these are controlled for in analyses [8].

Where there is a mismatch between individuals’ health-related literacy and numeracy skills and the demands of the health system, those with lower skills are at risk of poorer health. Low HL is associated with limited participation in screening for diseases, limited understanding of one’s illness or treatment plan, difficulties managing a chronic conditions such as diabetes mellitus, coronary health disease, heart failure, and asthma, poorer overall health status [5,9-14] and increased mortality [15].

Development of the evidence that links low HL and health has been facilitated by the use of measures of individual HL skills. The two most commonly used measures are the Test of Functional Health Literacy in Adults (TOFHLA) [16] and the Rapid Estimate of Adult Literacy in Medicine (REALM) [17]. Both TOFHLA and REALM have been validated for use in the UK [18,19]. However, both have disadvantages in use in research and practice. The length of time required for administration of the TOFHLA (22 minutes or more for the full version and up to 10 minutes for the shortened version) precludes its use in busy clinic settings and significantly increases the length of participant questioning if used in research. The REALM can be administered quickly (in less than 3 minutes) but, unlike the TOFHLA, does not test word comprehension or numeracy.

The Newest Vital Sign is a relatively new instrument, developed in the US and a validated predictor of health literacy, measuring both literacy and numeracy skills. Now described in more than 50 peer-reviewed journal articles, the NVS consists of a food nutrition label with six associated questions giving scores from 0 to 6 [20]. It is quick to administer (3 minutes), acceptable to patients, and accurately predicts health literacy levels when compared to the lengthier TOFHLA.

This study’s objectives were to undertake a process of cognitive testing with health practitioners, nutritionists, academics, and the public in the UK to (a) modify the NVS nutrition label to match the style and content of nutrition labels used in the UK and (b) modify the questions associated with the nutrition label so that terminology and language matched common language usage in the UK. This was followed by validation of the amended test (the “NVS-UK”) against the UK-validated version of the TOFHLA. The TOFHLA was chosen as the standard against which the NVS-UK would be validated as it tests comprehension, is an accepted standard test for health literacy [5] and was the standard against which the original US version of the NVS was validated [20].

## Methods

### Modifying the original NVS to develop the NVS-UK

The NVS nutrition label was adapted to conform to current UK food labeling practice and the questions were converted from US- to UK-style English. We did this with a web-based Delphi technique [21,22] that involved a panel of experts from clinical practice (medicine, nursing, pharmacy), public health, dietetics, research, adult education, and the food and drink industry. Recruitment of these experts was undertaken through the Health Literacy Group UK, a not-for profit organization that aims to raise the profile of health literacy as a remediable cause of health inequalities [23]. All Health Literacy Group UK members were invited by email to participate and all who expressed an interest were recruited. We asked these experts to assess nutrition labels used in the UK, to compare their content and layout to the nutrition label used on the original (US) version of the NVS, and to suggest modifications of the original NVS nutrition label to make it concordant with UK nutrition labels. We also asked them to make suggestions for modifying the wording of the questions that accompany the nutrition label. The intent of these modifications was to make the style of English in the questions correspond to common usage in the UK.

Participants then used a web-based Delphi technique to score the layout of the modified nutrition label and questions, ranking them on a 5-point scale in which 1 indicated complete disagreement that the nutrition label and questions were suitable for use in the UK, and 5 indicating complete agreement. Further modifications of the nutrition label and questions were made in response to these scores and suggestions from participants, and rounds of web-based scoring were continued until consensus was reached (i.e. all participants scoring 4 or 5) that the label and questions were suitable for use in the UK and no more suggestions for improvement were being made.

### Further Refinement of the NVS UK through Cognitive Testing in the Community

The nutrition label and questions were then tested for ease of understanding and acceptability by the public in a series of one-on-one interviews conducted by the market-research firm, Ipsos MORI. The individuals interviewed in this phase were residents of Lambeth borough in central London, an inner-city area with marked socio-demographic variation. Lambeth is the 14th most deprived of England’s 354 Boroughs, with a high proportion of residents from Black and Ethnic Minority (BEM) groups [24]. Recruitment was in–street in Lambeth, with the time of day and recruitment site varied to ensure a wide cross-section of participants. A multi-stage sampling procedure was undertaken in 4 cycles over 6 weeks enabling the research team to assure that at least 30% of participants were from groups likely to have lower health literacy, such as members of BEM groups, those with education qualification levels below the standard English educational achievement expected at age 16 (5 grades A-C in the English matriculation examinations (GCSE)) [25], and people from the lowest two social grades (grades D and E) on the National Readership Survey (NRS) social grading system. The NRS social grades are the standard for market research in the UK [26]; and are shown in Table 1. Prospective participants with low levels of spoken English were screened out of the research. The interviews took place in participants’ homes.

**Table 1:** National readership survey social grades

		**% of population (NRS 2010)**
A	Higher managerial, administrative and professional	4
B	Intermediate managerial, administrative and professional	22
C1	Supervisory, clerical and junior managerial, administrative and professional	29
C2	Skilled manual workers	21
D	Semi-skilled and unskilled manual workers	15
E	State pensioners, casual and lowest grade workers, unemployed with state benefits only	8

Available from http://www.nrs.co.uk/lifestyle.html.

Each participant was asked to complete the NVS UK questions, comment on question wording and label content and layout, and explain the processes they used to answer each question. They were asked to give feedback on the length of the survey and the clarity and difficulty of the questions.

This was an iterative process in which successive rounds of 15–20 interviews were carried out. Each round was followed by a review of the responses by project investigators and further modification of the NVS label and questions as indicated by interview results. Interview rounds continued until no more modifications were suggested. Participants in the cognitive interviews all gave informed consent and were offered a £25 voucher as compensation for their time.

The socio-demographic characteristics of the participants in the cognitive interviews were compared with local and national population characteristics using Office of National Statistics (ONS) 2007 mid-year estimates [27], ONS 2009 mid-year estimates [28] and 2001 UK census data [29].

#### Validation

Validation of the NVS-UK was assessed by comparing its performance to that of an accepted standard measure of health literacy, the TOFHLA [16,18], including the area under the Receiver Operating Characteristic (ROC) curve, and it’s correlation with education qualification attainment.

Data were collected on socio-demographic, lifestyle, and educational attainment in an interview that lasted 45–60 minutes. Age data were collected in 10-year age bands. The interview procedures were pilot tested with 20 Lambeth residents, following which the main validation survey was undertaken.

#### Instruments

The reference standard measure for HL used in this study, the TOFHLA, was developed from hospital materials and consists of a 50-item reading comprehension and 17-item numerical ability test, taking 22 minutes or more to administer. The reading items use a modified Cloze procedure, in which every 5th to 7th word in a passage is omitted and replaced with a blank space; the word to fit into each blank space is chosen from multiple-choice options. The numeracy items use prescription forms, clinic instructions, and medical insurance examples about which questions are asked requiring calculations. TOFHLA scores range from 0 to 100. A score of <60 represents inadequate health literacy; people with skills at this level are likely to experience the greatest barriers due to limited literacy and numeracy. A score of 60 to 74 represents marginal literacy; people scoring at this level may experience some difficulties understanding and using health information. Those scoring >75 have adequate literacy and are unlikely to experience problems arising from limited health literacy and numeracy skills.

Participants completed the NVS UK first followed by the UK-validated version of the TOFHLA.

#### Sample and recruitment

For validation against the TOFHLA, the sample size calculation was based on published reports on the validation of the original NVS, where correlation against the TOFHLA was 0.59 [20]. An unacceptable correlation was considered to be 0.3 (i.e. accounting for 9% of variance), and (based on previous data) a plausible correlation for purposes of power calculation was defined as 0.5 (or more). All correlations that could be shown to be significantly higher than 0.3 were regarded as acceptable. At least 250 subjects were required to give 90% power to detect such a difference.

The recruitment area for the validation stage was widened to include the London Borough of Southwark. Southwark is a borough neighbouring Lambeth with similar socio-demographic characteristics i.e. high levels of socio-economic deprivation and a high proportion of people from BEM groups [28]. Eligibility criteria were age 18 – 75 years, living at home, and ability to converse in English. We excluded potential participants if they were health care professionals (defined as people working in the National Health Service or private health care), did not live at home, had self-reported impaired vision (unable to read the test card), or were unable to hold a conversation with the interviewer due to cognitive impairment or inability to converse in English. Sampling aimed to recruit a sample reflecting the age, gender, NRS social grades and ethnic mix of Lambeth and Southwark. Recruitment was by postcode with interviewers assigned to clusters of postcodes with a high prevalence of residents fitting the desired recruitment profile. A total of 51 sample points were issued, with 7 interviews to achieve within each sample point. Interviewers knocked at the doors of potential recruits; if no-one eligible for the study was available or participation was declined, the interviewer went to the next address on their list. The interviews took place in participants’ homes with consent. Participants were given a £15 gift voucher in compensation for their time.

#### Data analysis

The principal analysis to determine the validity of the NVS-UK was to assess the correlation (Pearson r) between scores on the NVS and an accepted standard measure of health literacy, the TOFHLA [16,18] and by calculating the area under the receiver operating characteristic (ROC) curve. Validity was further assessed by the correlation (Pearson’s r) between the NVS and participants’ educational qualification attainment.

Optimal cut-off point(s) on the NVS UK for differentiating different levels of health literacy as identified by the TOFHLA were undertaken through calculation of the sensitivity and specificity for selected cut scores in the ROC analysis.

Statistical analyses were performed using STATA. V 11.2.

#### Ethics review

The cognitive testing and validation interviews were conducted by Ipsos MORI under the Market Research Society (MRS) Code of Conduct and Interviewer Quality Control Scheme (IQCS). Ethics approval for the study was granted by the London South Bank University Ethics Committee (ref UREC 1034). This project was exempt from NHS Research Ethics Approval as participants were not recruited from the NHS.

## Results and discussion

### Delphi survey and cognitive interviews

All Health Literacy Group UK members (n=254) were approached to participate by email; 28 volunteered to do so. The areas of interest and expertise of the expert panel is shown in Table 2.

**Table 2:** **Area of interest/expertise of Delphi expert panel**^**1**^

nursing	2
dietetics	4
nutritionist	1
public health	13
health (other please state)^2^	9
literacy language and numeracy	5
education practitioner / teacher	4
health literacy	20
research	13
food and drink industry	1
Other non-health (please specify)^2^	8

^1^ Participants could identify more than one area of expertise or interest.

^2^ Other (1 unless otherwise stated).

Vocational and independent living rehabilitation, Quality improvement & use and collection of patient stories, Health Psychology, Pharmacy (2), Health promotion, Health policy, Medical anthropology, Health systems, Lay perspectives, Patient involvement and public engagement.

Participants were from a wide range of health, psychology, education, patient and public involvement, and industry. The expert panel reached consensus (scoring for all questions 4 or 5 out of a maximum 5) on the format and contents of the amended label and questions after 3 rounds on the web-based Delphi survey. After five cycles of cognitive interviews in the community, involving 80 local residents and with modifications made based on participant feedback, all participants found the NVS acceptable. The socio-demographic characteristics of the participants of the cognitive interview stage are shown in Table 3.

**Table 3:** Cognitive testing to amend Newest Vital Sign (NVS) UK: Socio-demographic characteristics of participants

		**n**	**Sample %**	**Lambeth %**	**England %**
Gender^1^	Male	44	55.0	52.3	49.6
Age^1^	18-24	11	13.8	12.3	13.2
	25-34	19	23.8	35.0	18.1
	35-44	17	21.3	24.2	20.5
	45-54	13	16.3	14.2	18.8
	55-64	11	13.8	8.3	16.6
	65-75	9	11.3	6.0	12.8
Social grade^2^	ABC	42	52.5	71.9	67.6
	DE	38	47.5	28.1	32.4
BEM^3^		51	63.8	40.0	14.5
Sample		80	100		

^1^ Based on Office of National; Statistics (ONS) 2009 midyear estimates [28].

^2^ National Readership Society grades: Based on 2001 Census data (Adults aged 18–74) [29].

^3^ Black and Ethnic Minority: Based on ONS 2007 midyear estimates [27].

The final version of the NVS UK is shown in Table 4 (showcard) and Table 5 (accompanying questions and correct responses).

**Figure 1:**
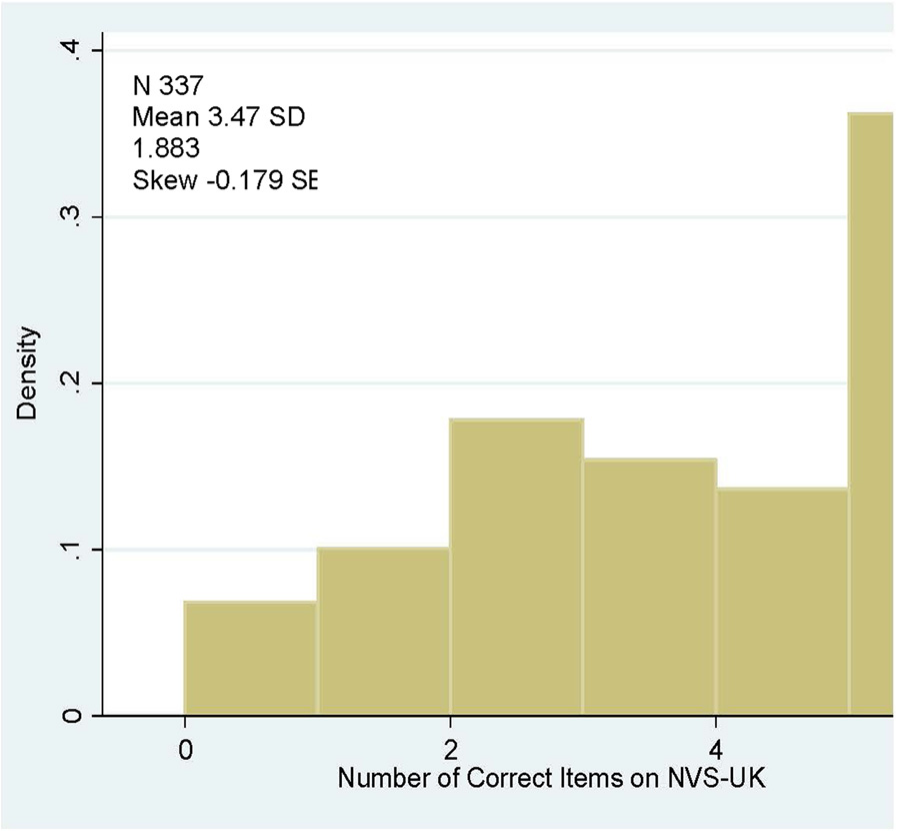
Distribution of scores NVS-UK.

**Table 4:** NVS Showcard

**Product Description: Ice Cream**		
Serving Size:	100ml	
Servings per container:	4	
**NUTRITIONAL INFORMATION**		
***TYPICAL VALUES***		***Per 100ml***
**Energy**		**1050 kJ**
		**250 kcal (calories)**
**Protein**		**4 g**
**Carbohydrate**		**30 g**
of which sugars		23 g
**Fat**		**13 g**
of which saturates		9 g
of which monounsaturates		0 g
of which polyunsaturates		3 g
of which trans fats		1 g
**Fibre**		**0 g**
**Sodium**		**0.05 g**

Ingredients: Cream, Skimmed Milk, Sugar, Whole Egg, Stabilisers (Guar Gum), Peanut Oil, Vanilla Extract (0.05%).

### Validation

A total of 337 participants were recruited for the validation study (Table 6). As planned, the sample included at least 30% representation from groups likely to have low literacy skills: 32% from social grades D and E, 36% with education below level 2, and 53% members of a BEM group. The high percentage of BEM participants reflected the ethnic mix of the local population.

**Table 5:** NVS UK questions and correct responses

**Question**	**Correct response**
1. How many calories (kcal) will you eat if you eat the whole container?	1,000 KCAL or 1,000 CALORIES
2. If you are advised to eat no more than 60 grams of carbohydrate for dessert, what is the maximum amount of ice cream you could have?	Two servings (or anything up to 2 servings) OR Half the container (or any amount up to half the container) OR 200 ml (or any amount up to 200 ml).
3. Imagine that your doctor advises you to reduce the amount of saturated fat in your diet. You usually have 42 g of saturated fat each day, some of which comes from one serving of ice cream. If you stop eating ice cream, how many grams of saturated fat would you be eating each day?	33 g
4. If you usually eat 2500 calories each day, what percentage of your daily calorie (kcal) intake will you get if you eat one serving of ice cream?	1/10 (one tenth) OR 10%
Imagine that you are allergic to the following substances: penicillin, peanuts, latex gloves, and bee stings.	
5. Is it safe for you to eat this ice cream?	No
If ‘No’ to Q5:	
6. Why not?	Because it contains peanut oil/peanuts/nuts OR Because you might have an allergic reaction
ASK IF answer to Q6 is ‘Because you might have an allergic reaction’:	
7. Why would you have an allergic reaction?	Because it contains peanut oil/peanuts/nuts

The final test subjected to validation consisted of a nutrition label and six questions, with one point awarded for each correct answer, giving a minimum score of 0 and a maximum score of 6. Total scores in this study ranged from 0 to 6, with a mean of 3.5 (Standard Deviation (SD) 1.8). The distribution of NVS UK scores is shown in Figure 1.

**Figure 2:**
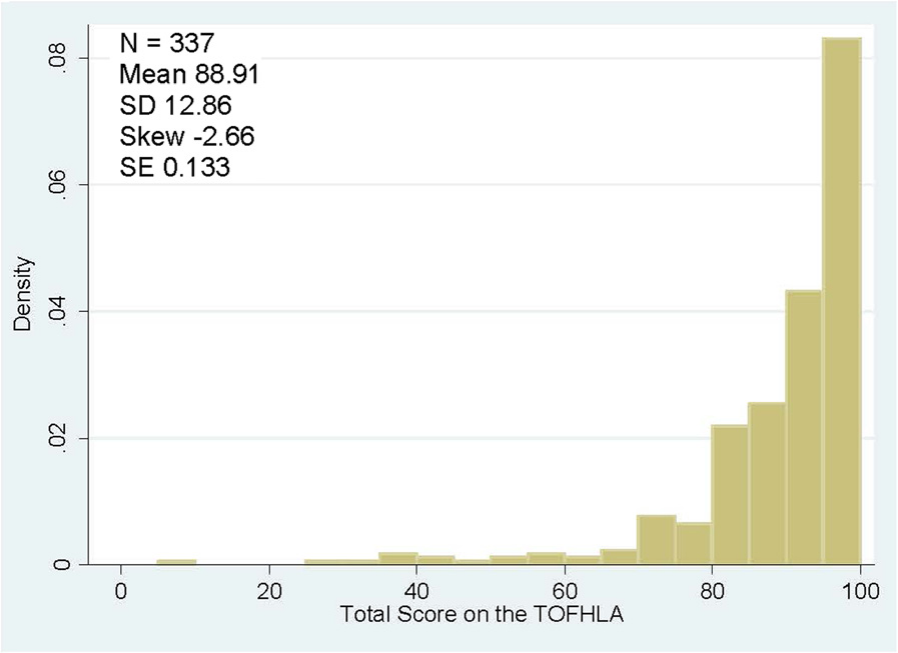
Distribution of scores TOFHLA (total score).

Total scores on the TOFHLA-UK ranged from 0 to 100 (mean = 88.9 SD 12.8). As reported previously, TOFHLA scores were skewed, with larger numbers of participants scoring at the higher end of the scale [20,30,31], higher scores indicate higher levels of health literacy. The distribution of total TOFHLA scores is shown in Figure 2.

**Figure 3:**
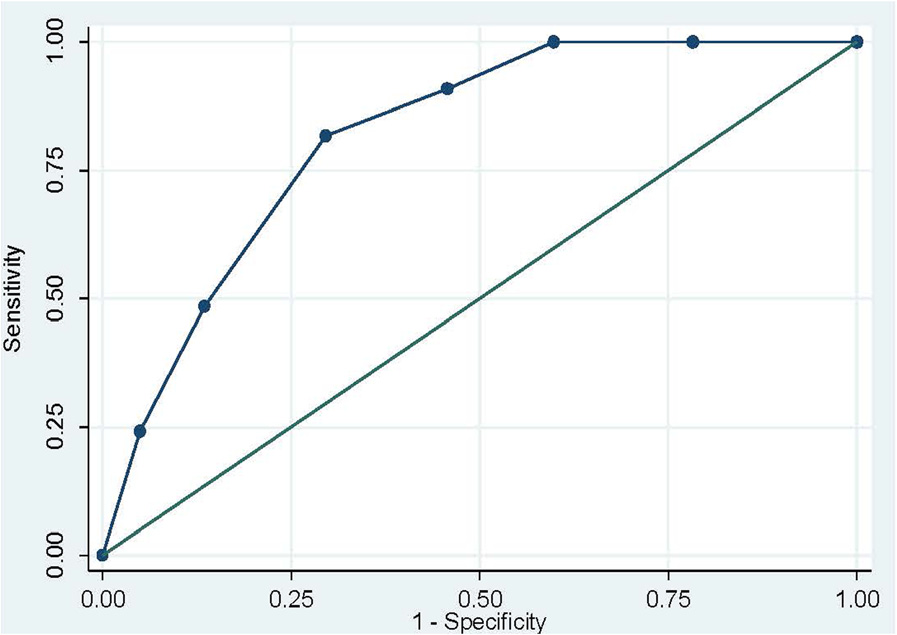
Receiver operating characteristics curve: ability of NVS-UK scores to predict TOFHLA scores.

The internal consistency of the NVS UK was high (Cronbach’s Alpha = 0.74).

The correlation against the reference standard TOFHLA was 0.49 on 332 observations (95% CI: 0.40 to 0.57), meaning that 24% (95% CI 16 to 32) of variance is accounted for; which can be deemed acceptable as it is significantly higher (P<0.001) than the unacceptable value of 0.30 set in the power calculation.

The area under the receiver operating characteristic (ROC) curve for predicting TOFHLA scores was 0.81 (Standard Error (SE) 0.0302, 95% confidence interval 0.76 to 0.88). This is shown in Figure 3.

The ROC analysis explored the sensitivity (true positive) and specificity (true negative) of different cut-off points for predicting adequate health literacy as defined by the TOFHLA. These are shown in Table 7. As expected, decreasing threshold levels reduced the likelihood of correctly identifying those individuals with adequate health literacy as defined by the TOFHLA (sensitivity) and increased the likelihood of correctly identifying people with TOFHLA scores below the ‘adequate’ threshold i.e. intermediate or low health literacy (specificity). An NVS-UK cut-off level of ≥ 4 would correctly identify all those with adequate health literacy as defined by the TOFHLA but would only identify 40% of those with health literacy levels below adequate as identified by the TOFHLA. A cut-off point of < 2 would, in contrast, only correctly identify 82% of those with adequate health literacy as defined by the TOFHLA, but would correctly identify 70% of those with lower health literacy as identified by the TOFHLA, The NVS-UK showed a low positive correlation with educational level (Pearson’s r=0.22). Although low, this was higher than the correlation with education levels of the TOFHLA literacy and numeracy subscales, and for the combined TOFHLA scores (Pearson’s r = 0.13, 0,16 and 0.09 respectively).

**Table 6:** Validation of NVS UK: Socio-demographic characteristics of participants

		**N**	**%**	**Lambeth and Southwark %**	**England %**
Gender^1^	Male	162	48.1	52.0	49.6
	Female	175	51.9	48.0	50.4
Age^1^	18-24	55	16.3	13.4	13.2
	25-34	89	26.4	33.2	18.1
	35-44	71	21.1	23.8	20.5
	45-54	64	19.0	14.9	18.8
	55-64	31	9.2	8.6	16.6
	65+	27	8.0	6.2	12.8
Social grade^2^	A-C	225	67	70	67.6
	D-E	109	32	30	32.4
	Unknown	3	.9		
Ethnicity^3^	White British	157	47	58	85
	Black and Ethnic Minority	177	53	42	15
	Not stated	3	0.9		
Education level^4^	Level 0 and 1	120	36	33	46
	Level 2 and above	217	64	62	47
	Unknown	0	0	5	6.9
Sample		337	100		

1. Based on Office of National; Statistics (ONS) 2009 mid year estimates [28].

2. National Readership Survey social grades: 2001 Census data [29].

3. Based on 2001 Census data [29].

4. Education level 2 equivalent to 5 passes at grade A-C in GCSE (English school matriculation) examinations, the standard expected at age 16 years [25].

**Table 7:** Optimal cut-off points for prediction of adequate health literacy levels on the TOFHLA

**Cut point**	**Sensitivity (true positive)**	**Specificity (true negative)**
6	100	0
≥ 5	100	22
≥ 4	100	40
≥ 3	91	54
≥ 2	82	70
≥ 1	48	87
0	24	95

### Discussion

We have modified the original NVS to develop a new version that is suitable for use in the UK. The NVS-UK has face validity, as it tests skills used in everyday life (i.e. understanding and interpreting information on a food nutrition label) – a factor that is likely to contribute to its acceptability to patients [32]. The instrument measures both text comprehension (literacy) and numeracy skills.

In addition, our analysis shows that the NVS-UK has good psychometric characteristics. Scores correlate well with a UK-validated version of the more complex and lengthy TOFLHA. Importantly, the area under the ROC curve of 0.81 was high, indicating high accuracy and, in fact, higher accuracy than many screening tests that are widely used in clinical practice [33-35]. The broader distribution and lesser skewness of the NVS scores across the population when compared with the TOFHLA (Figures 1 and 2) indicate a better ability to discriminate across a wider range of health literacy levels.

The ROC analysis identified optimal cut-off points for interpreting NVS-UK results in research or clinical practice. A score of 4 or more would correctly identify all those with adequate health literacy skills, a score of 2 – 3 would indicate intermediate health literacy skills, and a score of 0 – 1 would indicate low health literacy skills. These cut-off levels are the same as those found in validation of the original NVS. These values can be used in both research and clinical practice to identify individuals likely to have health literacy skills at those three levels.

It important to note, however, both the NVS and the TOFHLA are assessment tools that identify only certain aspects of the ‘cognitive and social skills needed by individuals as they access, understand, and use information in ways that promote and maintain good health’ [1,2]. Neither will measure the full range of skills needed to be ‘health literate’, or can assign a specific reading or numeracy level. Our study does show, however, that the NVS-UK is valid as a screening test that gives an accurate prediction of health literacy skills in comparison to the more complex and longer TOFHLA, and is likely to discriminate across a wider range of skills levels.

It should be noted that our study only included people able to converse in English. Further studies are required to determine potential use of the NVS-UK in people who have limited English skills. This may be facilitated by its translation into and validation in other languages. A validated Spanish version of the NVS is available [20] and the instrument has recently been translated into Dutch and Turkish, [36,37].

Finally, although the UK-NVS had a higher correlation with educational attainment than the TOFHLA or REALM, the correlation is nonetheless low. This low correlation was not unexpected as it is known that educational attainment is not a good predictor of literacy skills; many individuals have literacy skills well below what might be expected from the number of years of schooling they completed [38]. When health literacy skills need to be ascertained in research or practice, education level should not be used to make this determination. Rather, a direct measure such as the NVS-UK should be used.

## Conclusions

### Implications for research

The NVS-UK is a simple and accurate predictor of health literacy skills. Previous studies [20,32,39] show that it takes an average of 3 minutes to complete and can be administered by both clinical and non-clinical personnel. This, combined with its acceptability to patients [32] makes it an ideal measure to be used in surveys, cohort studies, and clinical trials in which health literacy may be a factor.

### Implications for practice

The NVS has been widely used in clinical practice to aid practitioners and practice managers in understanding the HL skills of their patient populations, and such assessments have been found acceptable to nearly all patients [40,41].

An interesting possibility is the potential use of HL assessment as a diagnostic tool when patients appear to be experiencing difficulties in understanding and managing complex conditions or adhering to medication or other treatment regimens, as HL is known to be a predictor of poor adherence [42-44]. Further research is required to investigate this.

### Summary

The speed, simplicity, validity and acceptability of the NVS-UK make it an ideal research tool to investigate the role of health literacy in health and illness. It also has a potentially valuable role in improving clinical practice and patient communication.

## Abbreviations

BEM: Black and ethnic minority;HL: Health literacy;NVS: Newest vital sign;ONS: Office of national statistics;REALM: Rapid estimate of adult literacy in medicine;ROC: Receiver operating characteristic;TOFHLA: Test of functional health literacy in adults

## Competing interests

The authors declare that they have no competing interests.

## Authors’ contributions

GR contributed to the development of the project protocol, chaired the project steering group, led on the Delphi rounds and led on writing the paper. NK led on obtaining funding for the project, and contributed to protocol development, and the project steering group. She led on data analysis and contributed to writing the paper. EO-N contributed to developing the protocol, the project steering group and writing the paper. PS provided supervision of the data analysis and interpretation and contributed to writing the paper. SB provided nutrition expertise and co-led on the design of the nutrition label and questions that form the NVS-UK. She contributed to the paper. BDW developed the original NVS, on which the NVS-UK was based. He contributed to the project protocol, and co-led on the design of the nutrition label and questions that form the NVS-UK. He contributed to the paper. All authors read and approved the final manuscript.

Full size electronic copies (pdf) of the NVS UK showcard, questions and administration instructions are available free of charge from the following url: http://www.healthliteracy.org.uk/newest-vital-sign-uk.

## Authors’ information

GR (corresponding author) is Professor of Health Disparities at London South Bank University, UK and Chair of the Health Literacy Group UK http://www.healthliteracy.org.uk.

NK is a Consultant Community Midwife at Guy’s and St Thmoas’ NHS Foundation Trust, London, UK.

EO-M is Head of Obstetrics at Guy’s and St Thomas’ NHS Foundation Trust, London UK.

PS is a non-clinical Senior Lecturer in Medical Statistics at King’s College London, UK.

SB is a Research Dietician at King’s College London, UK.

BDW is a Professor of Family Medicine at the University of Arizona, US.

## Pre-publication history

The pre-publication history for this paper can be accessed here:

http://www.biomedcentral.com/1471-2458/13/116/prepub
